# (3-Chloro­prop­yl)triphenyl­phospho­nium bromide

**DOI:** 10.1107/S1600536812042122

**Published:** 2012-10-13

**Authors:** Channappa N. Kavitha, Hemmige S. Yathirajan, A. S. Dayananda, Thomas Gerber, Eric Hosten, Richard Betz

**Affiliations:** a University of Mysore, Department of Studies in Chemistry, Manasagangotri, Mysore 570 006, India; b Nelson Mandela Metropolitan University, Summerstrand Campus, Department of Chemistry, University Way, Summerstrand, PO Box 77000, Port Elizabeth, 6031, South Africa

## Abstract

The title compound, C_21_H_21_ClP^+^Br^−^, is the bromide salt of a mixed aryl-alkyl phospho­nium cation. C–P–C angles span a range of 107.20 (10)–111.18 (10)°. The non-H atoms of the 3-chloro­propyl group adopt a staggered conformation [C–C–C–Cl torsion angle: −72.0 (3)°]. In the crystal, C—H⋯Br contacts connect the entities of the title compound into a double zigzag chain along *b*. These chains are linked into a supra­molecular layer lying parallel to (10-1) by C—H⋯π inter­actions.

## Related literature

For synthetic applications of phospho­nium salts in organic chemistry, see: Maercker (1965 ▶); Carruthers (1971 ▶); Minami *et al.* (1988 ▶). For related structures, see: Czerwinski & Ponnuswamy (1988*a* ▶,*b* ▶). For graph-set analysis of hydrogen bonds, see: Etter *et al.* (1990 ▶); Bernstein *et al.* (1995 ▶).
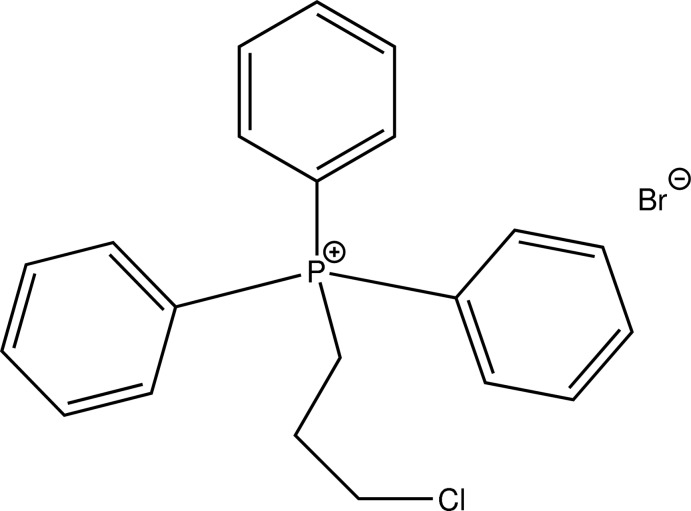


## Experimental

### 

#### Crystal data

C_21_H_21_ClP^+^·Br^−^
*M* *_r_* = 419.71
Monoclinic, 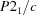

*a* = 11.0708 (2) Å
*b* = 10.0435 (2) Å
*c* = 17.5740 (4) Å
β = 104.973 (1)°
*V* = 1887.70 (7) Å^3^
*Z* = 4
Mo *K*α radiation
μ = 2.40 mm^−1^
*T* = 200 K
0.51 × 0.35 × 0.16 mm

#### Data collection

Bruker APEXII CCD diffractometer
Absorption correction: multi-scan (*SADABS*; Bruker, 2008 ▶) *T*_min_ = 0.324, *T*_max_ = 0.694
18046 measured reflections
4690 independent reflections
4202 reflections with *I* > 2σ(*I*)
*R*_int_ = 0.014

#### Refinement

*R*[*F* ^2^ > 2σ(*F* ^2^)] = 0.034
*wR*(*F* ^2^) = 0.107
*S* = 1.06
4690 reflections
217 parameters
H-atom parameters constrained
Δρ_max_ = 1.69 e Å^−3^
Δρ_min_ = −0.64 e Å^−3^

### 

Data collection: *APEX2* (Bruker, 2010 ▶); cell refinement: *SAINT* (Bruker, 2010 ▶); data reduction: *SAINT*; program(s) used to solve structure: *SHELXS97* (Sheldrick, 2008 ▶); program(s) used to refine structure: *SHELXL97* (Sheldrick, 2008 ▶); molecular graphics: *ORTEP-3 for Windows* (Farrugia, 1997 ▶) and *Mercury* (Macrae *et al.*, 2008 ▶); software used to prepare material for publication: *SHELXL97* and *PLATON* (Spek, 2009 ▶).

## Supplementary Material

Crystal structure: contains datablock(s) I, global. DOI: 10.1107/S1600536812042122/tk5158sup1.cif

Supplementary material file. DOI: 10.1107/S1600536812042122/tk5158Isup2.cdx

Structure factors: contains datablock(s) I. DOI: 10.1107/S1600536812042122/tk5158Isup3.hkl

Supplementary material file. DOI: 10.1107/S1600536812042122/tk5158Isup4.cml

Additional supplementary materials:  crystallographic information; 3D view; checkCIF report

## Figures and Tables

**Table 1:** Hydrogen-bond geometry (Å, °) *Cg*1 is the centroid of the C31–C36 ring.

*D*—H⋯*A*	*D*—H	H⋯*A*	*D*⋯*A*	*D*—H⋯*A*
C1—H1*B*⋯Br1^i^	0.99	2.82	3.703 (2)	149
C25—H25⋯Br1^ii^	0.95	2.89	3.751 (3)	151
C14—H14⋯*Cg*1^iii^	0.95	2.68	3.623 (3)	173

Symmetry codes: (i) 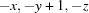; (ii) 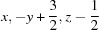; (iii) 
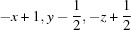
.

**Fig. 1.**
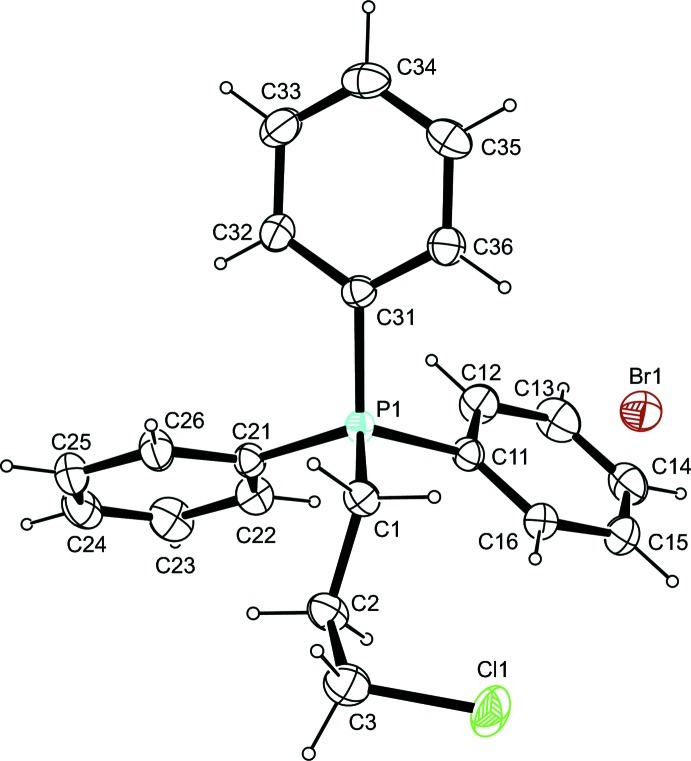
The molecular structure of the title compound, with atom labels and anisotropic displacement ellipsoids (drawn at 50% probability level).

**Fig. 2.**
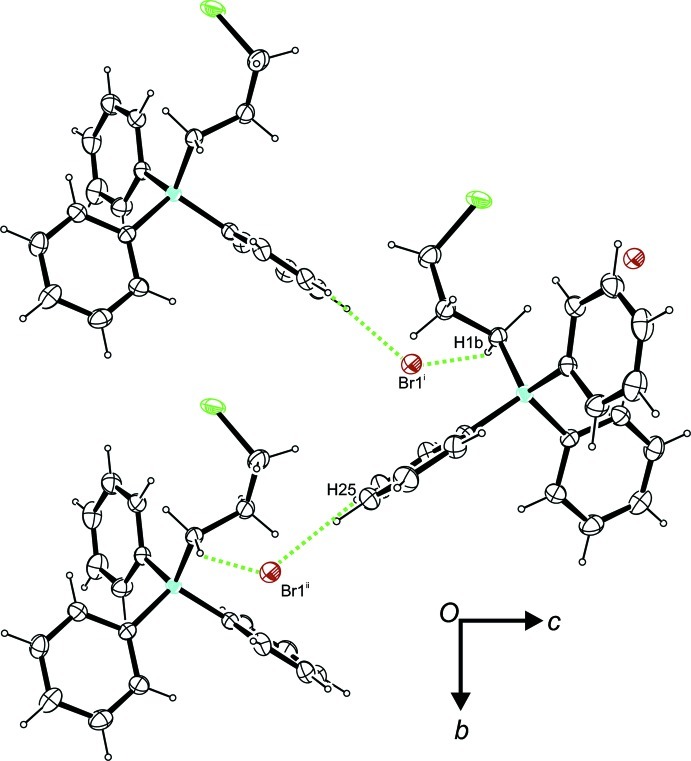
C–H···Br contacts, viewed along [-1 0 0]. Symmetry operators: i -x, -y + 1, -z; iix, -y + 3/2, z - 1/2.

**Fig. 3.**
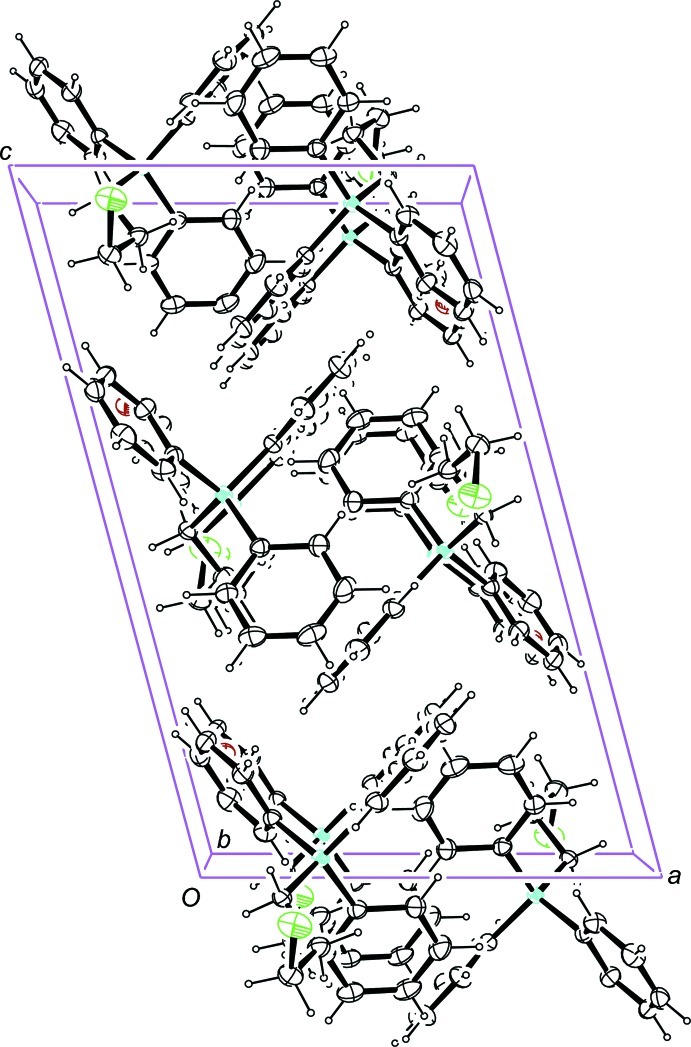
Molecular packing of the title compound, viewed along [0 1 0] (anisotropic displacement ellipsoids drawn at 50% probability level).

## supplementary crystallographic information

### Comment

Phosphonium salts are widely used in organic synthesis for the preparation of alkenes (Maercker, 1965; Carruthers, 1971) and are formed by alkylation of triaryl or trialkyl phosphines. Reports on (cycloalkylidenemethyl)triphenylphosphonium salts being used as versatile intermediate reagents have been published (Minami *et al.*, 1988). The crystal structures of several mixed alkyl-aryl phosphonium bromides have been reported such as (3-cyanopropyl)triphenylphosphonium bromide (Czerwinski & Ponnuswamy, 1988*a*) and (3-bromopropyl)triphenylphosphonium bromide (Czerwinski & Ponnuswamy, 1988*b*).

The phosphorus atom is coordinated tetrahedrally. The C–P–C angles span a range of 107.20 (10)–111.18 (10)° with the smallest angle found in between two phenyl groups and the largest angle in between a phenyl and the 3-chloropropyl group. The non-hydrogen atoms of the 3-chloropropyl group adopt a staggered conformation, the corresponding C–C–C–Cl angle is found at -72.0 (3)° (Fig. 1).

In the crystal, two C–H···Br contacts whose range falls by more than 0.1 Å below the sum of van der Waals radii of the corresponding atoms are observed. These are supported by a hydrogen atom of a phenyl group as well as a hydrogen atom of the methylene group directly bonded to the phosphorus atom. In terms of graph-set analysis (Etter *et al.*, 1990; Bernstein *et al.*, 1995), the descriptor for these contacts is *DD* on the unary level. Furthermore, a C–H···π contact stemming from one of the H atoms on a phenyl group is observed. Taking into account only the contacts that involve the bromide ion, the entities of the title compound are connected to a double zigzag chain along the crystallographic *b* axis. Metrical parameters as well as information about the symmetry of these contacts are summarized in Table 1. The shortest intercentroid distance between two aromatic systems was measured at 4.8882 (16) Å and is apparent between one of the phenyl groups and its symmetry-generated equivalent (Fig. 2).

The packing of the title compound in the crystal is shown in Figure 3.

### Experimental

The title compound was obtained as a gift sample from R. L. Fine Chem., Bengaluru, India. The compound was recrystallized from methanol by slow evaporation at room temperature and was used as such for diffraction studies.

### Refinement

Carbon-bound H atoms were placed in calculated positions (C—H 0.95 Å for aromatic carbon atoms, C—H 0.99 Å for methylene groups) and were included in the refinement in the riding model approximation, with *U*(H) set to 1.2*U*_eq_(C).

### Crystal data

**Table:**

C_21_H_21_ClP^+^·Br^−^	*F*(000) = 856
*M**_r_* = 419.71	*D*_x_ = 1.477 Mg m^−^^3^
Monoclinic, *P*2_1_/*c*	Melting point: 498 K
Hall symbol: -P 2ybc	Mo *K*α radiation, λ = 0.71073 Å
*a* = 11.0708 (2) Å	Cell parameters from 9926 reflections
*b* = 10.0435 (2) Å	θ = 2.4–28.3°
*c* = 17.5740 (4) Å	µ = 2.40 mm^−^^1^
β = 104.973 (1)°	*T* = 200 K
*V* = 1887.70 (7) Å^3^	Block, colourless
*Z* = 4	0.51 × 0.35 × 0.16 mm

### Data collection

**Table:**

Bruker APEXII CCD diffractometer	4690 independent reflections
Radiation source: fine-focus sealed tube	4202 reflections with *I* > 2σ(*I*)
Graphite monochromator	*R*_int_ = 0.014
φ and ω scans	θ_max_ = 28.3°, θ_min_ = 2.4°
Absorption correction: multi-scan (*SADABS*; Bruker, 2008)	*h* = −14→14
*T*_min_ = 0.324, *T*_max_ = 0.694	*k* = −12→13
18046 measured reflections	*l* = −23→23

### Refinement

**Table:**

Refinement on *F*^2^	Primary atom site location: structure-invariant direct methods
Least-squares matrix: full	Secondary atom site location: difference Fourier map
*R*[*F*^2^ > 2σ(*F*^2^)] = 0.034	Hydrogen site location: inferred from neighbouring sites
*wR*(*F*^2^) = 0.107	H-atom parameters constrained
*S* = 1.06	*w* = 1/[σ^2^(*F*_o_^2^) + (0.0596*P*)^2^ + 2.6325*P*] where *P* = (*F*_o_^2^ + 2*F*_c_^2^)/3
4690 reflections	(Δ/σ)_max_ < 0.001
217 parameters	Δρ_max_ = 1.69 e Å^−^^3^
0 restraints	Δρ_min_ = −0.64 e Å^−^^3^

### Fractional atomic coordinates and isotropic or equivalent isotropic displacement parameters (Å^2^)

**Table:**

	*x*	*y*	*z*	*U*_iso_*/*U*_eq_	
Br1	0.11226 (2)	0.37223 (2)	0.168258 (14)	0.02748 (9)	
Cl1	0.19533 (8)	0.23552 (6)	−0.03470 (4)	0.03790 (17)	
P1	0.27583 (5)	0.69448 (6)	0.03725 (3)	0.01760 (12)	
C1	0.1762 (2)	0.5615 (2)	−0.01109 (13)	0.0210 (4)	
H1A	0.1693	0.4937	0.0284	0.025*	
H1B	0.0914	0.5972	−0.0347	0.025*	
C2	0.2259 (2)	0.4953 (3)	−0.07590 (14)	0.0264 (5)	
H2A	0.2184	0.5587	−0.1200	0.032*	
H2B	0.3157	0.4743	−0.0545	0.032*	
C3	0.1561 (3)	0.3691 (3)	−0.10672 (16)	0.0309 (5)	
H3A	0.1774	0.3415	−0.1558	0.037*	
H3B	0.0650	0.3862	−0.1195	0.037*	
C11	0.4085 (2)	0.6285 (2)	0.10851 (13)	0.0204 (4)	
C12	0.4923 (2)	0.7181 (3)	0.15544 (15)	0.0292 (5)	
H12	0.4798	0.8112	0.1477	0.035*	
C13	0.5936 (3)	0.6713 (3)	0.21322 (17)	0.0354 (6)	
H13	0.6509	0.7321	0.2449	0.043*	
C14	0.6108 (2)	0.5356 (3)	0.22457 (15)	0.0351 (6)	
H14	0.6800	0.5034	0.2644	0.042*	
C15	0.5282 (3)	0.4463 (3)	0.17839 (16)	0.0324 (6)	
H15	0.5410	0.3533	0.1866	0.039*	
C16	0.4263 (2)	0.4921 (2)	0.12005 (14)	0.0251 (5)	
H16	0.3695	0.4308	0.0884	0.030*	
C21	0.3235 (2)	0.7892 (2)	−0.03666 (13)	0.0209 (4)	
C22	0.4486 (2)	0.8127 (3)	−0.03397 (15)	0.0272 (5)	
H22	0.5130	0.7795	0.0085	0.033*	
C23	0.4780 (3)	0.8854 (3)	−0.09427 (18)	0.0349 (6)	
H23	0.5630	0.9006	−0.0935	0.042*	
C24	0.3837 (3)	0.9355 (3)	−0.15517 (16)	0.0344 (6)	
H24	0.4046	0.9861	−0.1957	0.041*	
C25	0.2597 (3)	0.9131 (3)	−0.15792 (15)	0.0329 (6)	
H25	0.1957	0.9484	−0.1999	0.039*	
C26	0.2288 (2)	0.8389 (3)	−0.09917 (15)	0.0284 (5)	
H26	0.1436	0.8218	−0.1013	0.034*	
C31	0.1948 (2)	0.8024 (2)	0.08894 (13)	0.0199 (4)	
C32	0.1688 (2)	0.9347 (2)	0.06715 (15)	0.0267 (5)	
H32	0.1929	0.9704	0.0232	0.032*	
C33	0.1073 (3)	1.0143 (3)	0.10998 (16)	0.0318 (5)	
H33	0.0901	1.1049	0.0958	0.038*	
C34	0.0712 (2)	0.9608 (3)	0.17351 (16)	0.0305 (5)	
H34	0.0285	1.0151	0.2024	0.037*	
C35	0.0965 (2)	0.8299 (3)	0.19506 (15)	0.0295 (5)	
H35	0.0710	0.7944	0.2385	0.035*	
C36	0.1593 (2)	0.7495 (2)	0.15347 (14)	0.0256 (5)	
H36	0.1779	0.6595	0.1687	0.031*	

### Atomic displacement parameters (Å^2^)

**Table:**

	*U*^11^	*U*^22^	*U*^33^	*U*^12^	*U*^13^	*U*^23^
Br1	0.03111 (15)	0.02528 (14)	0.02542 (14)	0.00090 (9)	0.00617 (10)	−0.00151 (9)
Cl1	0.0561 (4)	0.0166 (3)	0.0364 (3)	−0.0049 (3)	0.0036 (3)	0.0049 (2)
P1	0.0175 (3)	0.0176 (3)	0.0169 (2)	−0.0003 (2)	0.00315 (19)	0.00100 (19)
C1	0.0201 (10)	0.0225 (11)	0.0196 (10)	−0.0031 (8)	0.0036 (8)	−0.0018 (8)
C2	0.0300 (12)	0.0267 (12)	0.0235 (11)	−0.0052 (10)	0.0090 (9)	−0.0035 (9)
C3	0.0325 (13)	0.0294 (13)	0.0291 (12)	−0.0046 (10)	0.0049 (10)	−0.0065 (10)
C11	0.0181 (10)	0.0239 (11)	0.0183 (10)	0.0016 (8)	0.0031 (8)	0.0008 (8)
C12	0.0278 (12)	0.0275 (12)	0.0288 (12)	−0.0031 (10)	0.0011 (10)	−0.0035 (10)
C13	0.0231 (12)	0.0500 (17)	0.0289 (13)	−0.0046 (12)	−0.0011 (10)	−0.0062 (12)
C14	0.0239 (12)	0.0552 (18)	0.0240 (11)	0.0134 (12)	0.0022 (9)	0.0040 (12)
C15	0.0348 (13)	0.0334 (13)	0.0285 (12)	0.0127 (11)	0.0074 (10)	0.0064 (10)
C16	0.0276 (11)	0.0246 (11)	0.0227 (11)	0.0028 (9)	0.0057 (9)	0.0016 (9)
C21	0.0229 (10)	0.0202 (10)	0.0203 (10)	−0.0016 (8)	0.0066 (8)	0.0016 (8)
C22	0.0247 (12)	0.0294 (12)	0.0278 (12)	−0.0042 (9)	0.0073 (9)	0.0000 (10)
C23	0.0334 (14)	0.0371 (14)	0.0381 (14)	−0.0124 (11)	0.0165 (12)	−0.0004 (11)
C24	0.0520 (17)	0.0277 (13)	0.0283 (12)	−0.0093 (12)	0.0191 (12)	0.0004 (10)
C25	0.0438 (15)	0.0317 (13)	0.0233 (11)	0.0032 (12)	0.0092 (11)	0.0059 (10)
C26	0.0279 (12)	0.0344 (13)	0.0232 (11)	0.0016 (10)	0.0072 (9)	0.0057 (10)
C31	0.0191 (10)	0.0197 (10)	0.0202 (10)	0.0001 (8)	0.0039 (8)	−0.0022 (8)
C32	0.0310 (12)	0.0228 (11)	0.0254 (11)	0.0034 (9)	0.0056 (9)	0.0023 (9)
C33	0.0332 (13)	0.0245 (12)	0.0350 (13)	0.0070 (10)	0.0037 (11)	−0.0022 (10)
C34	0.0210 (11)	0.0377 (14)	0.0311 (12)	0.0040 (10)	0.0033 (9)	−0.0111 (11)
C35	0.0264 (12)	0.0379 (14)	0.0264 (12)	−0.0042 (10)	0.0108 (9)	−0.0048 (10)
C36	0.0277 (12)	0.0236 (11)	0.0267 (11)	−0.0017 (9)	0.0092 (9)	0.0009 (9)

### Geometric parameters (Å, º)

**Table:**

Cl1—C3	1.818 (3)	C16—H16	0.9500
P1—C11	1.793 (2)	C21—C22	1.393 (3)
P1—C21	1.796 (2)	C21—C26	1.400 (3)
P1—C31	1.796 (2)	C22—C23	1.393 (4)
P1—C1	1.799 (2)	C22—H22	0.9500
C1—C2	1.539 (3)	C23—C24	1.383 (4)
C1—H1A	0.9900	C23—H23	0.9500
C1—H1B	0.9900	C24—C25	1.380 (4)
C2—C3	1.511 (3)	C24—H24	0.9500
C2—H2A	0.9900	C25—C26	1.386 (4)
C2—H2B	0.9900	C25—H25	0.9500
C3—H3A	0.9900	C26—H26	0.9500
C3—H3B	0.9900	C31—C32	1.392 (3)
C11—C16	1.392 (3)	C31—C36	1.398 (3)
C11—C12	1.398 (3)	C32—C33	1.391 (4)
C12—C13	1.386 (4)	C32—H32	0.9500
C12—H12	0.9500	C33—C34	1.388 (4)
C13—C14	1.384 (5)	C33—H33	0.9500
C13—H13	0.9500	C34—C35	1.377 (4)
C14—C15	1.384 (4)	C34—H34	0.9500
C14—H14	0.9500	C35—C36	1.390 (4)
C15—C16	1.392 (3)	C35—H35	0.9500
C15—H15	0.9500	C36—H36	0.9500
			
C11—P1—C21	111.13 (11)	C11—C16—C15	119.3 (2)
C11—P1—C31	107.20 (10)	C11—C16—H16	120.3
C21—P1—C31	108.92 (11)	C15—C16—H16	120.3
C11—P1—C1	110.32 (11)	C22—C21—C26	120.2 (2)
C21—P1—C1	108.10 (11)	C22—C21—P1	122.69 (18)
C31—P1—C1	111.18 (10)	C26—C21—P1	117.13 (18)
C2—C1—P1	112.15 (16)	C21—C22—C23	119.3 (2)
C2—C1—H1A	109.2	C21—C22—H22	120.4
P1—C1—H1A	109.2	C23—C22—H22	120.4
C2—C1—H1B	109.2	C24—C23—C22	120.1 (3)
P1—C1—H1B	109.2	C24—C23—H23	120.0
H1A—C1—H1B	107.9	C22—C23—H23	120.0
C3—C2—C1	112.3 (2)	C25—C24—C23	120.9 (2)
C3—C2—H2A	109.1	C25—C24—H24	119.6
C1—C2—H2A	109.1	C23—C24—H24	119.6
C3—C2—H2B	109.1	C24—C25—C26	119.8 (3)
C1—C2—H2B	109.1	C24—C25—H25	120.1
H2A—C2—H2B	107.9	C26—C25—H25	120.1
C2—C3—Cl1	111.20 (18)	C25—C26—C21	119.8 (2)
C2—C3—H3A	109.4	C25—C26—H26	120.1
Cl1—C3—H3A	109.4	C21—C26—H26	120.1
C2—C3—H3B	109.4	C32—C31—C36	120.3 (2)
Cl1—C3—H3B	109.4	C32—C31—P1	122.14 (18)
H3A—C3—H3B	108.0	C36—C31—P1	117.55 (18)
C16—C11—C12	120.0 (2)	C33—C32—C31	119.7 (2)
C16—C11—P1	121.69 (18)	C33—C32—H32	120.2
C12—C11—P1	118.23 (18)	C31—C32—H32	120.2
C13—C12—C11	120.1 (3)	C34—C33—C32	119.7 (2)
C13—C12—H12	119.9	C34—C33—H33	120.1
C11—C12—H12	119.9	C32—C33—H33	120.1
C14—C13—C12	119.7 (3)	C35—C34—C33	120.7 (2)
C14—C13—H13	120.2	C35—C34—H34	119.7
C12—C13—H13	120.2	C33—C34—H34	119.7
C13—C14—C15	120.5 (2)	C34—C35—C36	120.3 (2)
C13—C14—H14	119.7	C34—C35—H35	119.9
C15—C14—H14	119.7	C36—C35—H35	119.9
C14—C15—C16	120.3 (3)	C35—C36—C31	119.3 (2)
C14—C15—H15	119.8	C35—C36—H36	120.4
C16—C15—H15	119.8	C31—C36—H36	120.4
			
C11—P1—C1—C2	−79.46 (19)	C1—P1—C21—C26	54.5 (2)
C21—P1—C1—C2	42.2 (2)	C26—C21—C22—C23	−0.3 (4)
C31—P1—C1—C2	161.75 (16)	P1—C21—C22—C23	179.0 (2)
P1—C1—C2—C3	169.87 (18)	C21—C22—C23—C24	1.2 (4)
C1—C2—C3—Cl1	−72.0 (3)	C22—C23—C24—C25	−0.9 (4)
C21—P1—C11—C16	−118.7 (2)	C23—C24—C25—C26	−0.3 (4)
C31—P1—C11—C16	122.4 (2)	C24—C25—C26—C21	1.2 (4)
C1—P1—C11—C16	1.2 (2)	C22—C21—C26—C25	−0.9 (4)
C21—P1—C11—C12	64.1 (2)	P1—C21—C26—C25	179.8 (2)
C31—P1—C11—C12	−54.8 (2)	C11—P1—C31—C32	124.8 (2)
C1—P1—C11—C12	−176.03 (19)	C21—P1—C31—C32	4.5 (2)
C16—C11—C12—C13	0.4 (4)	C1—P1—C31—C32	−114.5 (2)
P1—C11—C12—C13	177.7 (2)	C11—P1—C31—C36	−54.4 (2)
C11—C12—C13—C14	−0.4 (4)	C21—P1—C31—C36	−174.73 (18)
C12—C13—C14—C15	0.4 (4)	C1—P1—C31—C36	66.2 (2)
C13—C14—C15—C16	−0.2 (4)	C36—C31—C32—C33	0.1 (4)
C12—C11—C16—C15	−0.2 (4)	P1—C31—C32—C33	−179.1 (2)
P1—C11—C16—C15	−177.39 (19)	C31—C32—C33—C34	−0.7 (4)
C14—C15—C16—C11	0.1 (4)	C32—C33—C34—C35	0.5 (4)
C11—P1—C21—C22	−3.6 (2)	C33—C34—C35—C36	0.3 (4)
C31—P1—C21—C22	114.3 (2)	C34—C35—C36—C31	−0.9 (4)
C1—P1—C21—C22	−124.8 (2)	C32—C31—C36—C35	0.7 (4)
C11—P1—C21—C26	175.69 (19)	P1—C31—C36—C35	179.96 (19)
C31—P1—C21—C26	−66.4 (2)		

### Hydrogen-bond geometry (Å, º)

Cg1 is the centroid of the C31–C36 ring.

**Table:**

*D*—H···*A*	*D*—H	H···*A*	*D*···*A*	*D*—H···*A*
C1—H1*B*···Br1^i^	0.99	2.82	3.703 (2)	149
C25—H25···Br1^ii^	0.95	2.89	3.751 (3)	151
C14—H14···*Cg*1^iii^	0.95	2.68	3.623 (3)	173

Symmetry codes: (i) −*x*, −*y*+1, −*z*; (ii) *x*, −*y*+3/2, *z*−1/2; (iii) −*x*+1, *y*−1/2, −*z*+1/2.
